# Location of Keratin-associated Proteins in Developing Fiber Cuticle Cells using Immunoelectron Microscopy

**DOI:** 10.4103/0974-7753.77512

**Published:** 2010

**Authors:** LN Jones, GE Rogers, N Rufaut, RD Sinclair

**Affiliations:** Department of Dermatology, University of Melbourne (St. Vincent’s Hospital), Fitzroy, Australia; 1 School of Molecular and Biomedical Science, University of Adelaide, Adelaide, Australia

**Keywords:** Fiber cuticle, immunogold, protein location, transmission electron microscopy

## Abstract

**Aims:**

To investigate the location of keratin-associated proteins (KAPs) in developing hair fiber cuticle cells using transmission electron microscopy with immunogold techniques and specific antibodies. Other studies were aimed at detecting the presence of cornified envelope proteins including involucrin and loricrin.

**Mateials and Methods:**

Polyclonal antibodies were produced in rabbits against peptides from KAPS 5.1, KAPS 10.1 ultra high-sulfur proteins.

**Results:**

The KAP proteins were found to form part of the developing exocuticle and a- layer. Cornified envelope proteins (involucrin and loricrin) were absent consistent with recent findings.

**Conclusions:**

The results have been discussed in terms of a revised model for fiber cuticle surface barriers including their role in fiber cuticle surface function.

## INTRODUCTION

The mammalian hair fiber cuticle (FCu) consists of a series of resistant barriers that protect the underlying cellular and molecular components of fibers from the hazards of the surrounding environment.[1–3] In sheep, the physicochemical properties of these various surface barriers (surface envelope, exocuticle) are of interest to the wool industry because of the need for improved fiber performance, particularly for developing competitive easy-care garments. Likewise the cosmetic industry is reaching for new hair-care products based on a fundamental understanding of the structure, composition and formation of the human hair fiber cuticle and its hydrophobic surface properties.

Fiber cuticle cells are the end-product of the terminal differentiation pathway of epidermally derived keratinocytes located at the base of the wool follicle. Throughout this process, cell organelles are degraded and replaced by a series of laminae termed the exocuticle and endocuticle encased within a membranous envelope. The exposed surface part of this envelope is assembled late in the developmental pathway when it replaces the original cytoplasmic membrane (plasma membrane) and consists of a layer of cross-linked protein coated with covalently bound lipids.[3] The resulting surface membrane thus effectively forms the resilient material that protects hair fiber cuticle cells. This inherent protection is most likely due to the formation of a unique network of isopeptide crosslinks between proteins [ε-(γ-glutamyl) lysyl bonds] catalysed through the action of a family of enzymes known as transglutaminases (TGase).[4] TGase 3 has been reported to be the major one present in the cuticle and the cortex.[5] These bonds further reinforce the disulfide proteins of the exocuticle.[6,7]

The protein composition of the fiber cuticle layers, endocuticle, exocuticle and putative surface envelope, is only partially understood despite its importance in determining hair surface properties.[8]

Concomitant with formation of the fiber cuticle surface, a series of novel and unique lipids assemble in the intercellular space between apposed fiber cuticle and inner root sheath (IRS) cuticle cells where they serve several functions. In particular the branched chain fatty acid (18-methyleicosanoic acid) becomes attached to the protein components to form covalent linkages (ester and/or thioester) to complete the assembly of the fiber cuticle surface membrane (FCUSM).[3] An understanding of the biochemical nature of crosslinks, lipids and proteins which comprise the FCUSM is fundamental to developing future strategies that could lead to novel modifications of fiber surface properties.

The aim of this paper was to use immune-electron microscopy with appropriate antibodies to identify proteins of the surface envelope and the exocuticule of the wool fiber cuticle.

## MATERIALS AND METHODS

### Sheep skin collection and processing

All animal experiments described in this paper were conducted in South Australia at field stations of the South Australian Research and Development Institute (SARDI) and the School of Molecular and Biomedical Science, University of Adelaide. Animal welfare and ethical procedures of the University of Adelaide and SARDI were followed. At Turretfield Research Station (SARDI), South Australia, skin biopsies were obtained from a normal Corriedale-Merino cross sheep and a sheep made transgenic by established methods[9] to express the sheep trichohyalin gene specifically in the wool fiber cortex (FCo). These specimens were dissected into pieces with sides approximately 1×1 mm and immersed in fixative solution containing 0.25% v/v glutaraldehyde, 4% w/v sucrose, 4% w/v paraformaldehyde in phosphate-buffered saline (PBS) for 90 minutes at room temperature (RT). The pieces were then washed in PBS containing 0.4% w/v sucrose (2x) for 5 minutes at RT and subsequently dehydrated through a graded series of ethanol/water solutions (50% ethanol/H_2_O, 2x for 1-hour period and 100% v/v ethanol 2x for 4 hours each). They were then pre-embedded in 1:1, 100% v/v ethanol/LR White Resin for 1 hour and transferred to 1:2, 100% v/v ethanol/LR White Resin (ProSciTech, Australia) for a further 1 hour. Embedding in LR White Resin was undertaken firstly for 1 hour at RT followed by overnight curing in gelatine capsules at 50°C.

### Mouse skin processing

Blocks of skin from 6-day old and 10-day old albino mice were fixed and embedded as for sheep skin.

### Production of anti-sheep antibodies

**Figure 1:**
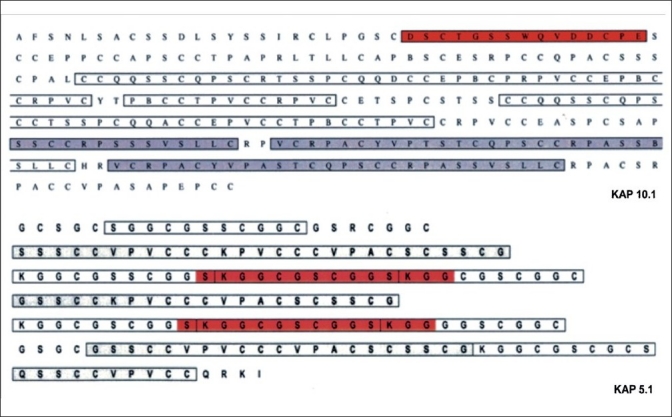
Amino acid sequences of sheep ultra-high sulfur proteins (KAP 5.1 and KAP 10.1). The peptides contained within each red box of the KAP 5.1 and KAP 10.1 protein sequences were used for production of anti-sheep antibodies used in immunolabelling experiments.[7]

Earlier work by MacKinnon *et al*.[6] has shown by *in situ* hybridization that mRNAs for ultra-high sulfur proteins are expressed in cells of the developing wool cuticle. From this finding and the use of EMBOSS programs, the antigenic peptide sequences were selected for antibody production.[10] The amino acid sequences of sheep ultra-high sulfur proteins (Keratin Associated Protein (KAP 5.1 and KAP 10.1)) are presented in Figure 1 where peptide sequences used for production of antibodies are included in the red boxed areas, respectively, as residues 61-74, repeating at 112-125 inclusive (KAP 5.1) and residues 25-39 inclusive (KAP 10.1). Hence a 14-mer peptide comprising SKGGCGSCGGSKGG (KAP 5.1) and a 15-mer peptide comprising DSCTGSSWQVDDCPE (KAP 10.1) were synthesized by AUSPEP (Parkville, VIC, Australia). These peptides were then used for production of anti-sheep antibodies in rabbits. Peptides (17 mg) were dissolved in PBS (500 μl, pH 7.2, in a glass vial) for conjugation to Keyhole Limpet Hemocyanin (KLH). Two additional solutions were prepared, one containing KLH (3 mg) dissolved in PBS (500 μl, pH 7.2) and the other 0.2% v/v aqueous glutaraldehyde fixative. The peptide solutions (50 μl) and the KLH solutions (70 μl) were subsequently mixed in a glass vial. These mixtures were cooled on ice for 5 minutes and shaken for a further 20 minutes at RT. A solution containing sodium borohydride (130 mg/ml) in PBS (pH 7.2) was prepared and an aliquot (20 μl) of this solution was added to each peptide mixture and placed in a shaker for 5 minutes. After cooling, the mixtures were kept at 4°C for a further 1 hour.[10]

To the cooled peptide mixtures, 240 μl of PBS (pH 7.2) and 500 μl of Freund’s Complete Adjuvant was added. These conjugates were vortexed thoroughly and the rabbits given two injections of 100 μl each over 3-4 weeks followed by booster injections at 2-week intervals but using incomplete adjuvant. Pre-immune blood (5-10 ml) for use as control serum was collected from two rabbits prior to injection of the peptide conjugates. All collected blood samples were allowed to clot for at least 2 hours. The sera were subsequently aspirated and centrifuged at 13,000 rpm for 5 minutes. The pellets were discarded and the supernatants kept at 4°C in the presence of 1% w/v sodium azide. The γ-globulin component in sera was collected on a Sepharose 4B column linked to Protein-A.[10] Attempts to estimate antibodies by immunoblotting were unsuccessful due to inadequate separation of proteins in the native form on electrophoretic gels. Consequently we have depended on the use of controls to assess the production and specificity of anti-sheep KAP 5.1 and KAP 10.1 antibodies (protein A-gold detection with and without pre-immune serum) within the immunogold technique. Experience indicates that estimation of titres are of little use since the main issue involves accessibility to antigenic sites *in situ*. We have prepared a positive control using the cross-species reactive anti-sheep trichohyalin antibody as a bench mark for selecting the optimum fixation conditions. This antibody is excellent for this purpose since it reacts specifically with trichohyalin granules of the IRS and medulla.[11]

Rabbit antibodies directed against mouse involucrin and loricrin peptides were purchased from Covance (Covance Research Products Inc., Richmond, CA). Immunogold procedures for detecting involucrin and loricrin in mouse follicle sections were essentially the same as these described below.

### Transmission electron microscopy and immunogold labelling

Serial, transverse ultra-thin sections were prepared from embedded skin specimens of sheep and mice using a diamond knife mounted on a Reichert OMU4 Ultracut. The sections were collected on carbon nitrocellulose-coated hexagonal nickel grids and monitored by optical microscopy at intervals for observing levels of fiber differentiation (optical sections 1-2μm, stained with 1% w/v gentian violet).

Grids were placed section side down onto drops of 50 mM glycine solution (pH 7.2) for 20 minutes and then blotted edge-on with filter paper. They were then transferred onto drops of 1% w/v ovalbumin (Sigma Grade V, chicken egg) in PBS (pH 7.2) for 20 minutes at RT. The grids were blotted edge-on and specimens were placed on a drop containing primary antibody diluted in 1% w/v ovalbumin in PBS (pH 7.2), as shown in Table 1.

**Table 1:** Antibodies used in this study together with recommended dilutions

Primary antibody	*Dilution*
Anti-sheep KAP 5.1	1:100
Anti-sheep KAP 10.1	1:100
Anti-mouse involucrin	1:10
Anti-mouse loricrin	1:10
Anti-sheep trichohyalin (positive control)	1:100
Pre-immune serum (negative control)	1:100

Grids were incubated for antigen/antibody reactions overnight at 4°C. After incubation, grids were placed onto drops (repeated 6x) of 1% w/v ovalbumin in PBS (pH 7.2) and finally blotted edge-on with filter paper. Grids were then transferred onto drops of the secondary reagent, Protein-A gold (PAG) (10 nm diameter) complex (Aurion, The Netherlands) diluted 1:100 with 1% w/v ovalbumin in PBS at pH 7.2. The grids were left for 60–90 minutes at RT and then washed in PBS (repeated 6x, 5 minutes each), followed by a thorough washing through a series of small beakers (3x) containing double distilled water.

Immediately after washing, grids were blotted edge-on with filter paper and stained in 4% w/v uranyl acetate (centrifuged 5000 g for 2 minutes) over a 10-minute period. The grids were then washed in double distilled water and drained edge-on with filter paper. In some cases sections were stained with Reynolds’ lead citrate.[12] Prior to staining the solution was centrifuged (5000 g for 2 minutes) and drops of stain placed under an atmosphere free of carbon dioxide by using sodium hydroxide pellets. Staining time was 5 minutes followed by washing in a series of beakers containing double distilled water.

Grids were examined in a Philips CM 10 transmission electron microscope operating at 100 kV and images were collected by digital processing.

## RESULTS

**Figure 2:**
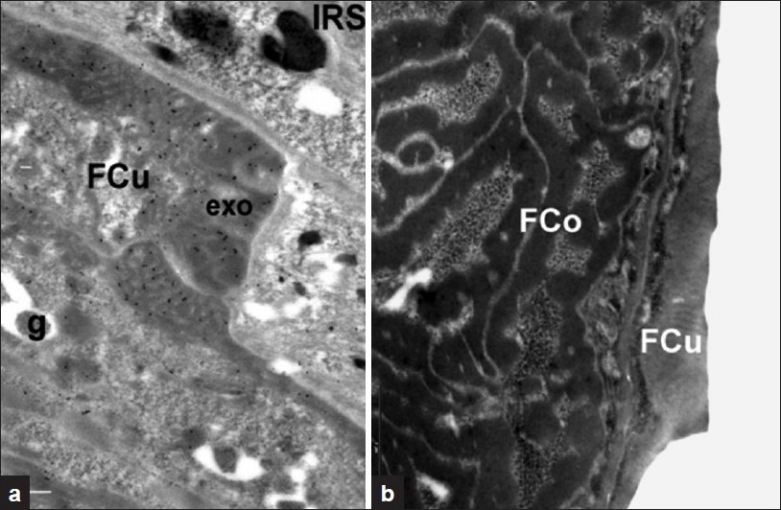
(a) Transverse section of differentiating wool follicle cuticle cells (FCu) after incubation with anti-sheep KAP 5.1 antibodies and Protein A-Gold (PAG). Labelling is predominately located in presumptive exocuticle (exo) and associated precursor granules (g) where present, and is absent in the IRS. Sections stained with uranyl acetate. Bar equals 100 μm; (b) Wool follicle section after incubation with pre-immune serum (negative control) showing the absence of PAG labelling in both FCu (cuticle) and FCo (cortical) cells. Section stained with uranyl acetate. Bar equals 1 μm

**Figure 3:**
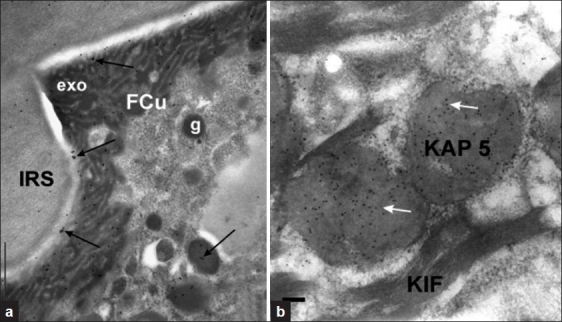
(a) Transverse section of differentiating wool FCu after incubation with anti-sheep KAP 10.1 and PAG. Labelling is predominately located in presumptive exocuticle (exo) and associated precursor granules (g) where present, and is absent in the IRS. There is also significant labelling in the fiber cortex indicating cross-reaction with related KAPs. Sections stained with uranyl acetate. Bar equals 1 μm; (b) Micrograph showing wool follicle section from a transgenic sheep (KAP 5.1). Incubation with anti-sheep KAP 5.1 purified post-immune rabbit serum demonstrates specific reaction with the KAP 5.1 protein inclusions found in these sheep. Section stained with uranyl acetate. Bar equals 0.1 μm

**Figure 4:**
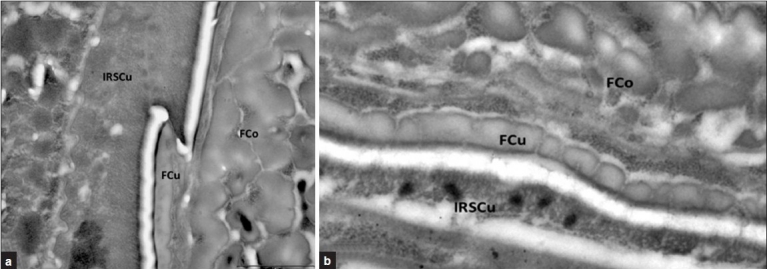
(a) and (b) Transverse section of mouse FCu after incubation with anti-mouse loricrin and anti-mouse involucrin and PAG. No antibody binding was observed in both cases. Section stained with uranyl acetate. Bar equals 1 μm

**Figure 5:**
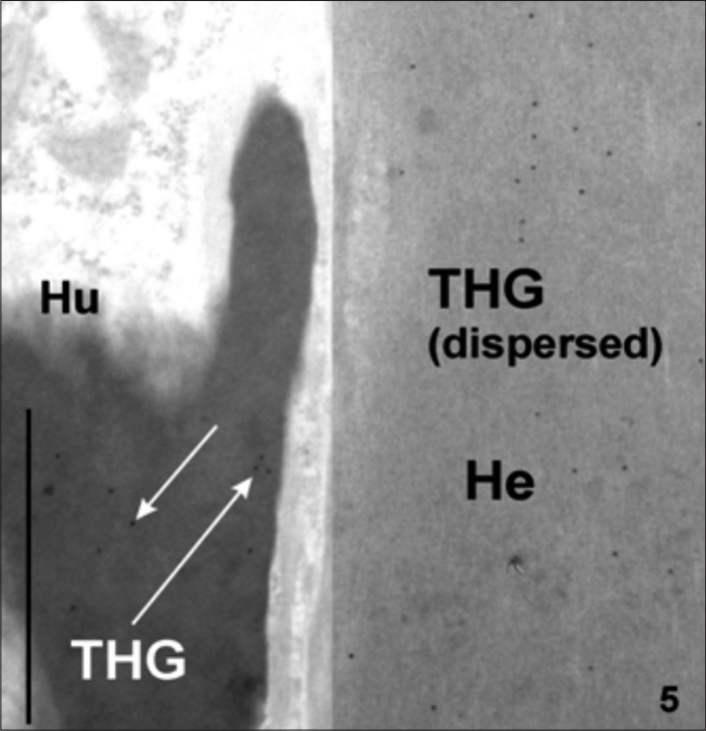
Wool follicle incubated with anti-sheep trichohyalin antibodies and PAG. These reactions demonstrated that trichohyalin was specifically labelled in trichohyalin granules (THG) in Huxley’s layer of the IRS but also when the trichohyalin was dispersed as a matrix in the Henle cell (He). Section stained with uranyl acetate. Bar equals 1μm

The micrographs shown in Figures 2–5 are examples of serial sections at various levels of the wool follicle probed with the various antibodies. Controls employed rabbit anti-sheep trichohyalin antibody as a positive control and pre-immune serum from rabbits was used as a negative control. We assessed the specificity of antibodies raised in rabbits against the ultra-high sulfur proteins by comparing post-inoculation sera with pre-immune serum using the immunogold technique. Pre-immune control serum [Figure 2b] indicated absence of PAG and therefore no evidence for non-specific reactions on wool follicle sections. Further, in order to examine the specificity of the KAP antibodies the KAP 5.1 antibody was tested with follicles from the skin of a transgenic sheep that was over-expressing the KAP 5.1 gene directed to express in the wool cortex by a cortex-specific promoter [data unpublished but see Bawden.[9]] In the presumptive cortex of these transgenic wool follicles, KAP 5.1 protein was deposited in high abundance with the appearance of inclusion bodies that reacted with the KAP 5.1 antibody as can be seen in [Figure 3b].

Another test of the efficacy of the methods used has been shown in Figure 5, where anti-sheep trichohyalin (positive control) antibody was bound specifically to trichohyalin granules in IRS cells of Huxley’s layer (Hu). In Henle’s layer (He) the granules have formed an interfilamentous matrix and consequently labelling is dispersed throughout the protein of the Henle layer.

In Figure 2a KAP 5.1 labelling can be seen located in a wool follicle transverse section. The micrograph shows regions of the wool follicle, including the developing fiber cuticle (FCu) and IRS at an early stage of wool fiber differentiation. Labelling is clearly observed in the developing exocuticle (exo) of FCu cells and precursor granules (g). Occasional labelling is evident in the cytoplasmic region of FCu cells such as the developing endocuticle but is absent in IRS cells.

The micrograph presented in Figure 3a shows follicle sections labelled with KAP 10.1 antibodies predominantly located in the developing exocuticle (exo) of FCu. Labelling is evident also in the precursor granules (g) shown arrowed. The IRS and trichohyalin granules (THG) contain a few labelled gold particles. It is important to note that the macrofibrils in the fiber cortex were found to also bind the KAP 5 and KAP 10 antibodies (see PAG labelling Figure 3a) indicating cross-reaction.

In experiments using loricrin and involucrin antibodies applied to sections of mouse hair follicles there was no binding to the fiber cuticle indicated by the absence of gold particles [Figures 4a and b].

## DISCUSSION

Previous studies over many years have indicated that the cuticle surface of hair and wool are rich in sulfur-containing proteins.[1,3,7] Although it has been suspected that KAP may be implicated in the fiber surface these moieties have never been identified. Given our aims in this study to characterise the fiber cuticle surface composition we can now provide direct evidence for the presence of KAP 5.1 and KAP 10.1 using immuno-electron microscopy techniques. In like manner we have used these techniques to further explore for the presence of envelope proteins in the cuticle and surface layers. However involucrin and loricrin have not been detected in this study confirming an earlier histochemical study.[13]

Given that a limited number of proteins have been confirmed as components of the fiber cuticle we suggest that the formation and assembly of the membrane does not appear to be a multi-stage process resembling the formation of the epidermal surface complex.[14] Since it is known that the surface layers of the cuticle are cross-linked it would appear that the KAP 5 and 10 proteins could become joined by the action of the cuticle TGase 3.[5] The hair fiber cuticle and outermost surface may have significantly different protein mixtures together with other minor as yet unknown proteins and the modes of formation may also be unique. The cuticle and particularly the outer surface proteolipid layers are difficult to study as they are highly inert and insoluble consequently only few techniques such as that used in the present study are available to characterize the various proteins and lipids located at the wool fiber surface.

A consistent observation in these studies has been that the antibodies (anti-KAP 5.1 and anti-KAP 10.1) were also bound to matrix (high-sulfur) proteins in the developing cortex of wool follicle cells. A possible explanation is that repeating sequences of high and ultra high-sulfur proteins are responsible, for example, a repeating pentapeptide found in numerous high sulfur proteins is well known.[7,15]

Zhan *et al*.[16] compared the amino acid content of the Allworden membrane[17] with those of cornified envelope (CE) proteins of keratinocytes[4] and by applying multiple linear regressions attempted a quantitative estimate of loricrin and involucrin contents in the membrane. The present results indicate that their conclusions are untenable. Thus the challenge remains to identify the protein structure of the cell envelope of the wool fiber surface. In that respect it is of importance to note that several keratin intermediate filament (KIF) proteins have been show to be present in human hair cuticle including a new finding of K80 and K80.1.[18] However, it is not known exactly where the KIFs are in the three main layers of the cuticle cell. The innermost layer of the cuticle, the endocuticle, is generally regarded as consisting of cytoplasmic remnants remaining after differentiation of the cuticle cell. The presence of a defined molecule, S100A3, a calcium-binding protein, in the endocuticle[19] is of interest in relation to cuticle properties.

The process of fixation using aldehydes can markedly influence the accessibility of epitopes through increased protein cross-linking thus masking and reducing the available epitopes. We consider that further examination to locate keratinocyte envelope proteins is warranted using cryosubstitution instead of aldehyde methods for preserving native antigenic sites of proteins *in situ* before a definitive conclusion can be made about a cornified envelope in the hair cuticle surface. In addition freeze substitution methods[20] could also increase the labelling potential of anti-mouse loricrin and involucrin in mouse hair follicle sections. Cryostudies are particularly important, leaving the possibility that these envelope proteins are absent in the fiber cuticle cell surface layers.

Further, using brief enzymes treatments of sections could also be useful in unmasking antigenic sites since they can be concealed in condensed protein structures such as the keratin proteins of wool fibers and follicles.

## CONCLUSIONS

**Figure 6:**
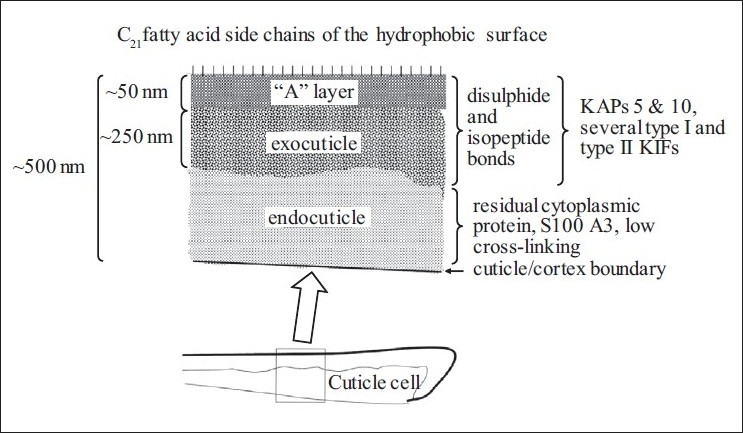
Diagrammatic representation of proposed model showing the fiber cuticle chemical components and their locations within the ultrastucture

Antibodies have been raised in rabbits directed against sheep ultra-high sulfur peptides derived from the KAP 5.1 and KAP 10.1 proteins. These antibodies have been used in immunoelectron microscopy studies to determine the locations of KAP 5.1 and KAP 10.1 *in situ*, in wool follicle sections. The results indicate that ultra-high sulfur proteins are located in the developing exocuticle. Parallel studies aimed at observing location of the cornified envelope proteins, involucrin and loricrin in the developing fiber cuticle surface were unsuccessful and these confirmed the recent findings of other authors. The present understanding of the protein composition of hair cuticle is summarised in Figure 6.

Characterisation of the wool fiber cuticle and surface is of fundamental interest to the wool industry. Future technologies will aim to modify surface properties to improve hair cosmetic appearance and aspects of wool processing.
